# A Simple Alternative Method for Preservation of 2-Methylisoborneol in Water Samples

**DOI:** 10.3390/ijerph15051015

**Published:** 2018-05-18

**Authors:** Chun-Cheng Fan, Yi-Ting Chiu, Tsair-Fuh Lin

**Affiliations:** Department of Environmental Engineering, National Cheng Kung University, Tainan City 701, Taiwan; ufoglove@hotmail.com (C.-C.F.); ostrich_0121@hotmail.com (Y.-T.C.)

**Keywords:** 2-methylisoborneol, sodium hypochlorite, preservation, potassium permanganate

## Abstract

2-Methylisoborneol (2-MIB) is one of the most commonly observed taste and odor (T&O) compounds present in drinking water sources. As it is biodegradable, a preservation agent, typically mercury chloride, is needed if the water is not analyzed right after sampling. Since mercury is a toxic metal, an alternative chemical that is cheaper and less toxic is desirable. In this study, two chemicals commonly used in water treatment processes, chlorine (as sodium hypochlorite) and KMnO_4_ (potassium permanganate), are studied to determine their feasibility as preservation agents for 2-MIB in water. Preservation experiments were first conducted in deionized water spiked with 2-MIB and with chlorine or permanganate at 4 and 25 °C. The results indicate that 2-MIB concentrations in the water samples spiked with both chemicals remained almost constant within 14 days for all the tested conditions, suggesting that oxidation and volatilization did not cause the loss of 2-MIB in the system. The experiments were further conducted for three different reservoir water samples with 30–60 ng/L of indulgent 2-MIB. The experimental results demonstrated that preservation with permanganate may have underestimated the 2-MIB concentration in the samples as a result of the formation of manganese dioxide particles in natural water and adsorption of 2-MIB onto the particles. Chlorine was demonstrated to be a good preservation agent for all three tested natural waters since oxidation of 2-MIB was negligible and biodegradation was inhibited. When the residual chlorine concentrations were controlled to be higher than 0.5 mg/L on the final day (day 14) of the experiments, the concentration reduction of 2-MIB became lower than 13% at both of the tested temperatures. The results demonstrated that sodium hypochlorite can be used as an alternative preservation agent for 2-MIB in water before analysis.

## 1. Introduction

Taste and odor (T&O) is an important issue for drinking water in many countries [1,2,3,4,5,6,7]. Among the many T&O compounds reported in the literature, 2-methylisoborneol (2-MIB) and trans-1,10-dimethyl-trans-9-decalo (geosmin) are the two most common chemicals detected in drinking water systems [6]. These compounds have caused customer complaints for decades [8]. Although they are of no concern to human health, they may raise customer suspicions related to water safety [9]. Therefore, detection and treatment of these compounds in drinking water are required so the water quality can be improved and the complaints of consumers can be reduced.

In drinking water sources, geosmin and 2-MIB can be produced from various microorganisms, including cyanobacteria [10,11], actinomycetes [12], fungi [13], and myxobacteria [14]. Although the contribution of actinomycetes and other microorganisms on geosmin and 2-MIB in source water cannot be neglected, cyanobacteria are generally considered as the major source of the two compounds. Many genera of cyanobacteria, including *Anabaena*, *Lyngbya*, *Oscillatoria*, *Phormidium*, *Planktothricoides*, *Planktothrix*, and *Pseudanabaena*, have been reported to be the producers of geosmin and/or 2-MIB [2,15,16,17,18,19]. Removing the two T&O compounds from drinking water is a challenge for water authorities internationally. In particular, consumers experience the two compounds as a musty, earthy odor at levels as low as 5–10 ng L^−1^ [20]. Therefore, the treatment methods for these compounds must be very effective [20]. Depending on the cyanobacteria strains, growth status, and environmental conditions, the odorants may either be contained within the cells (cell bound) or released into the water in a dissolved form [21]. These metabolites, particularly in their dissolved (extracellular) forms, have been shown to be somewhat unresponsive to conventional water treatment [22,23]. Therefore, understanding the ratio of intracellular and dissolved concentrations of the odorants is also important for water utility companies.

Analysis of geosmin and 2-MIB is commonly achieved by headspace solid phase microextraction (HS-SPME) coupled with gas chromatograph and mass spectrometry (GC-MS). In practical cases, water samples with geosmin and 2-MIB are taken in the field, including water sources and finished water, stored in amber glass vials with no headspace, and stored at 4 °C before analysis. As suggested in the APHA Method 6040 (2014), the samples should be analyzed within 14 days after the sampling. However, if the samples cannot be analyzed in 3 days, it is suggested that 10 mg L^−1^ of mercuric chloride be added into the sample to arrest biological degradation (APHA et al., 2014). Several studies have demonstrated bacterial degradation of 2-MIB even under low-temperature conditions in natural waters [24,25,26,27]. A few bacterial strains capable of degrading 2-MIB in water have been isolated from water treatment plants and reservoirs [24,26,28]. Therefore, if no preserving agent, such as mercury chloride, is added, biodegradation is expected to occur in samples after longer storage periods, leading to underestimation of 2-MIB and geosmin concentrations in the measurement.

In water treatment plants, chlorine and permanganate are commonly used for oxidation and/or disinfection. Studies [22,23,29,30] have demonstrated that 2-MIB is resistant to these two chemicals [29,30,31,32]. Less than 10% 2-MIB and geosmin were removed during chlorination and permanganation, at a chlorine dose = 2–10 mg Cl_2_/L and permanganate = 10 mg/L. Although the toxicity of the two chemicals is expected to be lower than that of mercury chloride, they are bactericides and may inhibit the biodegradation effect of 2-MIB in water samples. Because chlorine and permanganate do not destroy the two odorants, these two oxidants may be used as alternative preservation agents for 2-MIB and geosmin in water samples. In this study, the feasibility of the two alternative preservers for the analysis of 2-MIB in water samples is examined. The effect of temperature, oxidant type, and oxidant doses on the preservation and analysis of 2-MIB in deionized and natural waters are systematically studied, and the appropriate method obtained is reported.

## 2. Materials and Methods

### 2.1. Experiments

During the experiment, 2-MIB concentration was examined in three different environments: artificially spiked 2-MIB in deionized water and naturally present 2-MIB in both lake water and reservoir water. The samples were prepared in duplicate with two sets of variables, at temperatures of 4 °C and 25 °C, and with chlorine and permanganate added, in which the chlorine concentration was 2–10 mg/L and 10 mg/L for permanganate during 14 days of continuous analysis. The samples were analyzed using SPME (solid-phase microextraction) to extract the 2-MIB onto the fibers, and then gas chromatography coupled with a mass spectrometry was used to determine the 2-MIB concentration.

Natural water samples were collected from three water reservoirs, including the Agondan (AGD) Reservoir in Kaohsiung City, the Zengwen (ZW) Reservoir in Chiayi City, and the Nanhua (NH) Reservoir in Tainan City, Taiwan, ROC. The samples were collected at the water-intake points of the reservoirs for the experiment and were continuously analyzed 14 days after sample collection.

Preservation was performed with sodium hypochlorite (Merck Co., Darmstadt, Germany), expressed as Cl and potassium permanganate (KMnO_4_, Merck Co., Germany), in 60 mL amber glass vials, with Telflon^@^ caps (Thermo, Rockwood, TN, USA). The experimental processes were terminated by adding 20 µL of 10% (by weight) solution of sodium hyposulfite (J.T. Baker, Tokyo, Japan) into the vials. To determine the concentration of residual chlorine in the reactors, a water-quality analyzer (Hach DR3900, Loveland, CO, USA) was used. For the measurement of permanganate concentration, the Standard Method 4500-KMnO_4_ was used. Manganese dioxide powder (Sigma, Supelco Co., St. Louis, MO, USA, technical activated >90%) was used to study the adsorption of 2-MIB in the permanganate oxidation system. A standard solution of 2-MIB was obtained at a concentration of 100 µg/mL in methanol (Sigma, Supelco Co., Bellefonte, PA, USA), while the internal standards for both 2-isopropyl-3-methoxy pyrazine (IPMP) and 2-isobutyl-3-methoxy pyrazine (IBMP) were 100 µg/mL (Sigma, Supelco Co., USA). All the standards were stored at −20 °C before use. The standard solutions were then diluted with methanol to produce standard stock solutions. Calibration working solutions were prepared from the standard stock solutions to make the desired concentrations using deionized water from a Milli-Q ultrapure water purification system (Bedford Corp., Bedford, MA, USA).

### 2.2. Analysis Methods

The analytical method for 2-MIB was modified from the headspace solid-phase microextraction (HS-SPME) method of Lin et al. [6,31] and Hung et al. [33]. A 30/50 mm DVB/CAR/PDMS (No. 57348-U) fiber from Supelco (Bellefonte, PA, USA) coupled with a fiber holder (No. 57330-U, Supelco) was employed for the extraction and desorption of all the samples. The fiber was outgassed by baking at 270 °C for 0.5 h at the inlet of a gas chromatograph (GC) prior to its use for 2-MIB analysis. Twelve-milliliter amber glass vials with Teflon-silicone septa were used for the 2-MIB extraction. A sample volume of 4.5 mL, containing 1.54 g of added sodium chloride to saturate the solution, was used for analysis.

The sample vial was placed on a magnetic stirrer and agitated at 320 rpm. A fixed quantity of 2-isopropyl-3-methoxy pyrazine (IPMP) and 2-isobutyl-3-methoxy pyrazine (IBMP) (100 ng/L) was added to all the water samples and a blank, and calibration standards served as the internal standards. The extraction temperature was controlled at 80 °C using an aluminum heating block (Shinko Electronic Co., Taipei, Taiwan). For the extraction of the analytes, the SPME fiber was placed in the headspace of the sample vial for a predetermined time span, typically 30 min. After 30 min of exposure, the SPME fiber was injected into the GC for 2 min to desorb the trapped analytes.

A GC (Agilent 7890, Santa Clara, CA, USA) equipped with a mass spectrometer (MS) (Agilent 5975) was employed for the separation and quantification of the 2-MIB. The MS was operated in a positive chemical-ionization mode with methane (99.9995%, Yun Shan, Tainan, Taiwan) as the reagent gas to quantify the compound. A 30 m × 0.25 mm I. D. × 0.25 µm thick film HP-5MS column (Agilent) was used for separation of the T&O compounds. The sample transfer line to the mass spectrometer and the MS ion source were set at 260 °C and 250 °C, respectively. The injection port of the GC was installed with a 0.75-mm-inner-diameter SPME liner, operated in splitless mode, with the port heated to 260 °C. The GC oven temperature was initially held at 45 °C for 2 min, increased to 260 °C at a rate of 10 °C/min, and then held at 260 °C for 2 min. The total run time was 25.5 min. The carrier gas was ultrahigh-purity helium (99.9995%, Yun Shan, Taiwan) at a constant flow of 1.5 mL/min.

## 3. Results and Discussion

### 3.1. Impact of Chlorine on 2-MIB Concentration in Deionized Water

**Figure 1:**
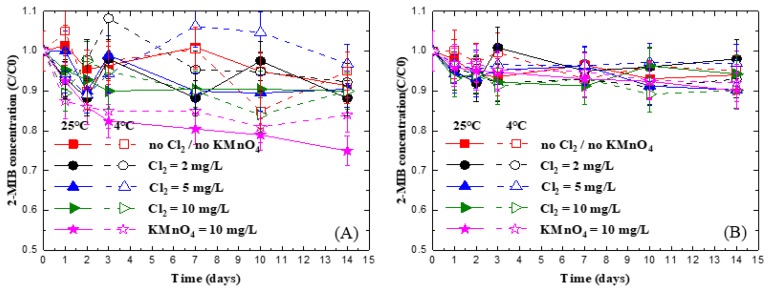
Changes of 2-MIB concentration in deionized water samples preserved with chlorine and permanganate at two temperatures, where initial 2-MIB concentration is (**A**) 20 ng/L and (**B**) 100 ng/L.

Figure 1 shows the effect of the chlorine dosages on the concentration changes in the 2-MIB in deionized water spiked with 100 and 20 ng/L of 2-MIB and stored at 4 and 25 °C. As shown in the figure, under all testing conditions, within 14 days, the 2-MIB concentrations were only changed by 5%–16%, suggesting that chlorine and permanganate are not able to destroy 2-MIB at the tested dosages. Lalezary et al. [22] studied the destruction of 2-MIB and geosmin by chlorine, permanganate, and two other oxidants. Their results indicated that under 10 mg/L dosages, only 10% of the 2-MIB was destroyed, which is similar to our observation. In addition to the effect of oxidation, in the current experimental setup, other loss mechanisms for 2-MIB, such as volatilization and biodegradation [32], were also insignificant. 

**Figure A1:**
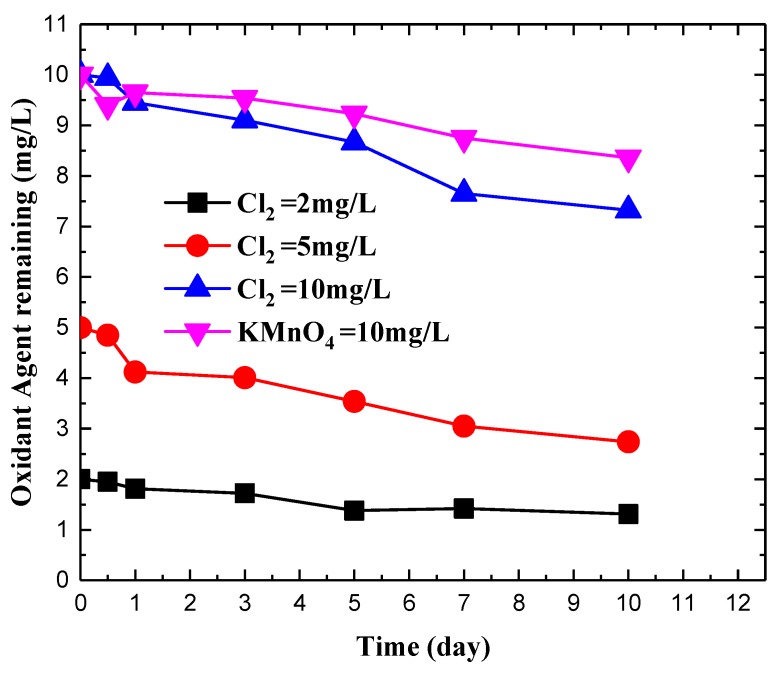
Residual chlorine concentrations for the preservation experiments of 2-MIB (initial concentration = 100 ng/L) in deionized water.

In the current study, deionized water was used and was expected to be free from or low in bacteria. Although 2-MIB is known to be biodegradable [27], biodegradation was expected to be negligible, leading to the results of almost constant 2-MIB concentrations in the samples through the experimental period. Another reason to lower 2-MIB biodegradation in the system is the addition of oxidants. Both chlorine and permanganate are known to be able to inactivate bacteria [33]. In the current experimental systems, the residual chlorine and permanganate (shown in Appendix Figure A1) within the experimental time remained relatively high, all >1 mg/L. Considering the unfavorable growth conditions due to high oxidant concentrations, the bacteria concentration present in the water was therefore expected to be even less, and low biodegradation in the system was also predicted.

Since both permanganate and chlorine have been shown to have the potential to be used as chemical preservatives for 2-MIB in deionized water, i.e., low reaction with 2-MIB and no biodegradation in the samples, the two oxidants were further tested for preservation of 2-MIB.

### 3.2. Impact of Permanganate on 2-MIB Concentration in Natural Water

**Figure 2:**
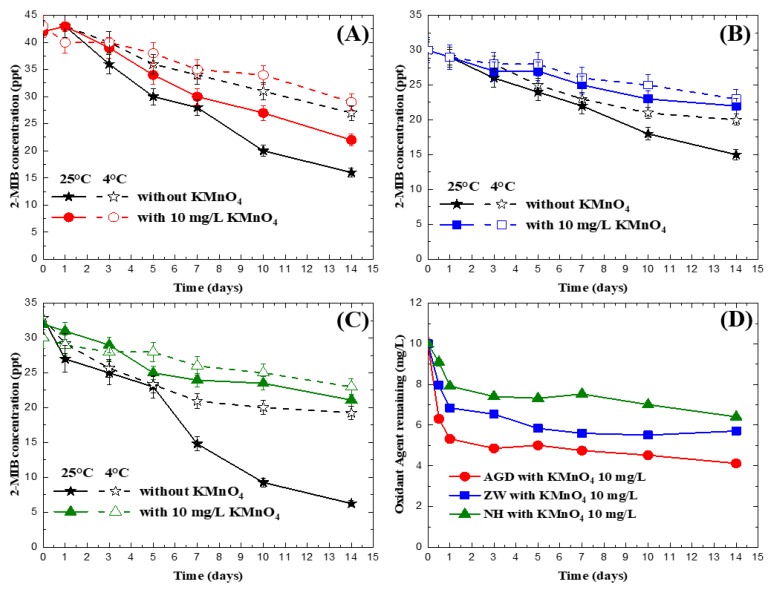
Changes of 2-MIB concentration in natural water samples preserved with permanganate, where (**A**) is for the AGD reservoir; (**B**) is for the ZW reservoir; (**C**) is for the NH reservoir; and (**D**) is for permanganate (initial conc. = 10 mg/L).

In the permanganate preservation experiments in natural water, three sources of water with the presence of 2-MIB—water from the NH, AGD and ZW reservoirs—at two temperatures, 4 and 25 °C, were tested with permanganate doses at 10 mg/L, for which the results are shown in Figure 2. It is noted in the figure that the control experiments represent samples with no addition of the chemical. As expected, 2-MIB concentrations were reduced significantly, by 26.1%–55.4%, for all the three tested waters within 14 days, which was likely due to biodegradation, as discussed in the previous section. For the preservation experiments with permanganate in the natural waters, unlike the results for deionized water where there was only <10% of reduction in 2-MIB concentrations with 14 days of preservation with 10 mg/L of permanganate, a 15%–26% and 23%–50% was found for the 2-MIB for the three tested natural waters at 4 °C and 25 °C, respectively. As shown in Figure 1 and in a report from Lalezary et al. [22], permanganate was not able to oxidize 2-MIB effectively. 

In addition, in the current experimental system, the loss of 2-MIB was not due to volatilization, as demonstrated in the control experiments (Figure 1). Since permanganate concentrations in the reactors were higher than 3.9 mg/L throughout the experiments (Figure 2D) and permanganate is a bactericide [34], it is expected that biodegradation would not be an important mechanism related to concentration reduction. Permanganate is known to form manganese dioxide (MnO_2_) particles after oxidation [35], and therefore the adsorption of 2-MIB onto the MnO_2_ was suspected to have caused the concentration change in 2-MIB in the system. To clarify the interaction between manganese dioxide and 2-MIB, a set of adsorption experiments were conducted. The experiments were similar to those used for the oxidation experiments except that 1 mg/L MnO_2_ and 100 ng/L 2-MIB were used, and the adsorption time was 14 days. 

**Figure 3:**
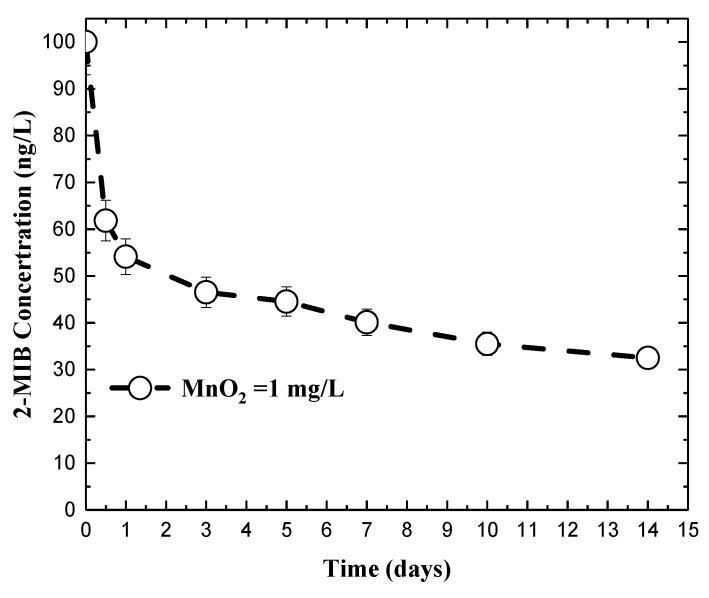
Change of 2-MIB concentration (100 ng/L) in deionized water with presence of manganese dioxide (1 mg/L).

Figure 3 illustrates the concentration changes in 2-MIB in the adsorption experiments. In the figure, the filtrate represents the samples filtered through a 0.3 μm-fiberglass filter (Advantec GF-75, Tokyo, Japan) before analysis, while the total represents the samples analyzed without filtration. The figure demonstrates that 2-MIB was reduced by 55.8% and 67.5% after reaction for 14 days for the samples with and without filtration, respectively. The results suggest that the MnO_2_ particles, both larger and smaller than 0.3 μm, were able to adsorb 2-MIB and cause a reduction in 2-MIB concentration. In the preservation experiments in natural water, 3.59–5.88 mg/L of permanganate were transformed into MnO_2_ in natural water (Figure 2D), approximately equal to 1.2–1.9 mg/L if based on stoichiometric calculation. Although the initial 2-MIB concentration between the permanganate preservation experiments in natural water (Figure 2) and the MnO_2_ adsorption experiments (Figure 3) were different, both experiments proved that removal of 2-MIB could be attributed to adsorption onto MnO_2_ in the two systems. For the permanganate preservation experiments in deionized water (Figure 1), less than 0.2 mg/L of MnO_2_ was expected to form, leading to lower adsorption of 2-MIB on MnO_2_. Therefore, the concentration reduction was not observed for 2-MIB. In natural water systems, many components, such as natural organic matters, algae metabolites, and reduced substance [36,37], may react with permanganate and form MnO_2_, causing adsorption of 2-MIB in the water. Based on this observation, therefore, permanganate cannot serve as a preservation chemical for 2-MIB analysis in water.

### 3.3. Impact of Chlorine on 2-MIB Concentration in Natural Water

Similar to permanganate, chlorine was further tested as a preservation agent for 2-MIB in natural water using the same natural water samples and the same temperatures. However, for the chlorination experiments, three chlorine doses, 2, 5, and 10 mg/L, were tested. In addition, before the analysis, all the samples were separated into two subsamples, one being filtered through a 0.3 μm-fiberglass filter to represent the extracellular 2-MIB, and the other being used for total 2-MIB (cell bound plus extracellular).

**Figure 4:**
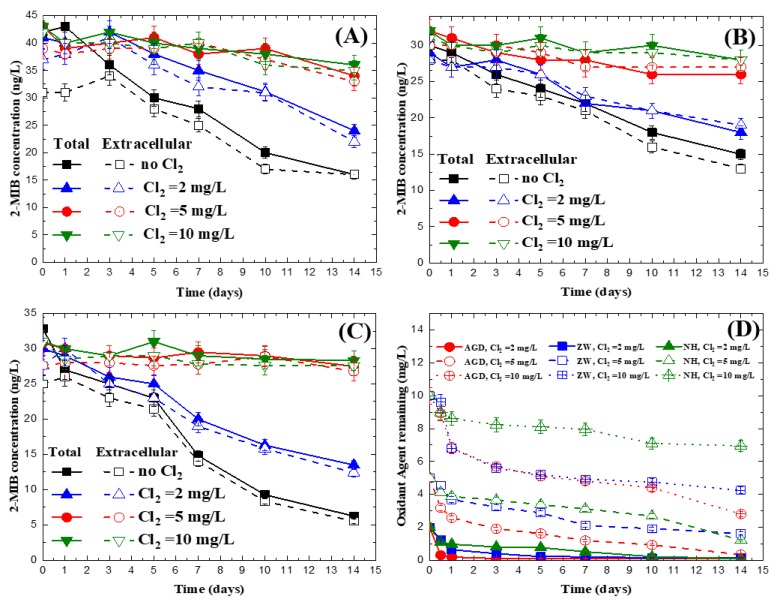
Changes of 2-MIB concentration in natural water preserved with chlorine and stored at 25 °C, where (**A**) is for the AGD reservoir; (**B**) is for the ZW reservoir; (**C**) is for the NH reservoir; and (**D**) is the residual chlorine concentrations.

**Figure A3:**
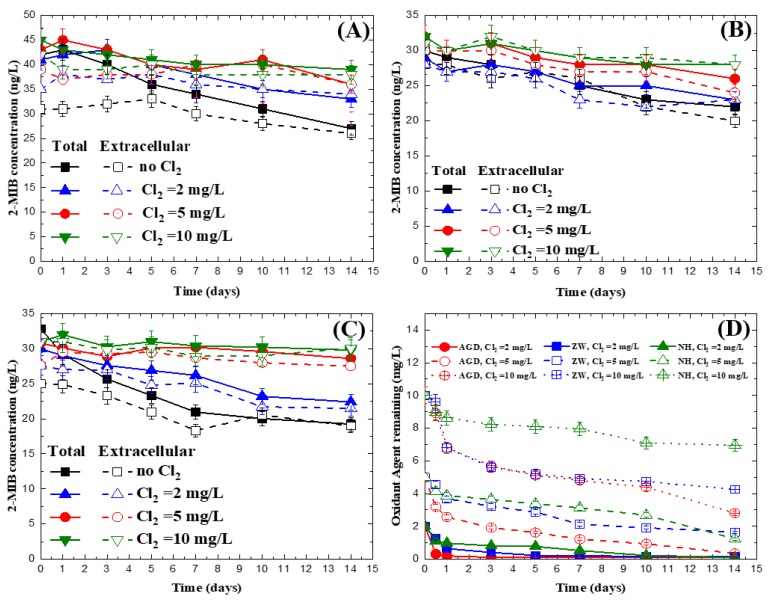
Changes of 2-MIB concentration in natural water preserved with chlorine and stored at 4 °C, where (**A**) is for the AGD reservoir; (**B**) is for the ZW reservoir; (**C**) is for the NH reservoir; and (**D**) is the residual chlorine concentrations.

Figure 4 and Figure A3 show the effect of temperature and chlorine dosage on preservation of 2-MIB in the three tested natural water samples at 25 °C and 4 °C, respectively. The figures clearly demonstrate that for the three natural water samples tested, the 2-MIB concentration was reduced by more than 20% after 14 days at both temperatures if chlorine dosages were at or below 2 mg/L. For those with chlorine dosages of 5 or 10 mg/L, 2-MIB concentrations only changed by <13% during the 14 days of experiments for all the cases tested. As shown in Figure 4D and Figure A3D, when the chlorine dosage was at 2 mg/L, the residual chlorine concentrations were all below 1 mg/L after the first day of the experiment and were almost equal to 0 after 3–5 days. However, for chlorine dosages at 5 and 10 mg/L, in all cases, residual chlorine was observed to be >1.5–2.0 mg/L in all the tested cases. Chlorine is known to be a very good bactericide, and reports [38] have shown that if the residual concentration is higher than 0.5 mg/L, bacterial growth is significantly suppressed. Therefore, it is reasonable to find that in these tested natural water samples, 2-MIB concentrations remained almost constant if the chlorine dosages were enough to maintain a residual chlorine concentration >1.5 mg/L.

Figure 4 and Figure A3 also demonstrate that a higher preservation temperature (25 °C) (Figure 4) led to higher reduction in the 2-MIB concentration, i.e., 37.9%–55.0% after 14 days, as compared with those at 4 °C (Figure A3), which ranged from 20.6% to 25.3%. Because bacteria prefers to grow at approximately 25 °C [39], it is reasonable to observe higher biodegradation of 2-MIB at that temperature. In the figure, it can also be seen that there are some differences between the total and extracellular 2-MIB concentrations for the three tested reservoir waters at day 0. Because the differences between the total and extracellular samples represent those bound with cells, it can be seen that at day 0, about 26.1%, 13.3%, and 23.9% of the 2-MIB was cell bound as calculated from the samples with no addition of chlorine. When the chlorine dosage was increased, the ratio of cell-bound 2-MIB decreased at day 0. Tung et al. [30] studied the interaction of chlorine and cyanobacteria cells and showed that cells may be damaged after contact with chlorine, causing the release of intracellular metabolites. Our observation of increasing chlorine dosages causing greater release of 2-MIB is consistent with the finding of their study. After 5 days of reaction, as shown in the figure, the total 2-MIB concentrations were almost the same as their corresponding extracellular 2-MIB concentrations, suggesting that all the cyanobacteria cells were lysed and that all the 2-MIB was released into the water. 

To further quantify the degradation of 2-MIB in the preservation experiments, the data of the three natural water cases (Figure 4) were simulated with a first-order reaction model. As shown in Table A1, the rate constants decreased with increasing chlorine concentrations added to the samples. Although the data may not be perfectly fitted by the model, the rate constants may provide a quick estimation of the degrees of degradation. For the cases of no chlorine addition, the rate constants were between 5.04 × 10^−2^ day^−1^ and 1.21 × 10^−1^ day^−1^. The rate constants decreased to 2.89 × 10^−2^ day^−1^ to 6.27 × 10^−2^ day^−1^, 9.79 × 10^−4^ day^−1^ to 1.95 × 10^−2^ day^−1^, and 5.65 × 10^−3^ day^−1^ to 1.21 × 10^−2^ day^−1^, for the cases of chlorine = 2, 5, and 10 mg/L, respectively. The results clearly showed that adding chlorine significantly slowed down the rates of degradation. 

**Figure A4:**
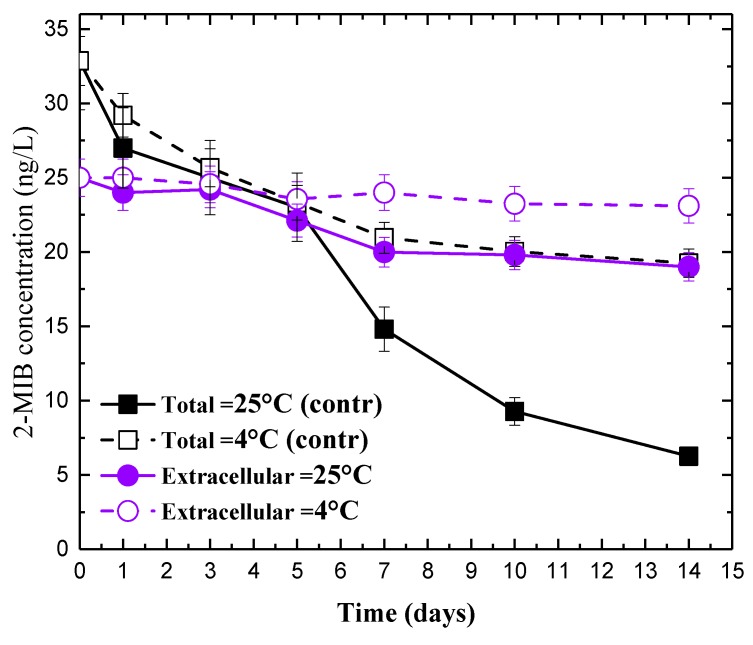
NH reservoir water sample with a 0.3 μm-fiberglass filter before the preservation experiment.

To test the applicability of filtration as an alternative to rule out biodegradation effects for 2-MIB, another set of experiments was conducted for the samples of NH water. The samples were filtered through 0.3 μm-fiberglass filters before the preservation experiments, which were used for filtering out the indulgent bacteria in the water. Figure A4 shows that after filtration, the 2-MIB concentration remained almost constant at 4 °C after 14 days, suggesting that in the samples, the degrading bacteria were filtered out. However, for other samples, including the extracellular samples stored at 25 °C and the samples with cells present, a reduction in 2-MIB >20% was observed. Because in natural water, 2-MIB may be present in both cell-bound and dissolved phases, filtration is not feasible if both cell-bound and extracellular 2-MIB are to be determined.

## 4. Conclusions

The experimental results for the deionized water show that both sodium hypochlorite at 2–10 mg/L and potassium permanganate at 10 mg/L may preserve 2-MIB in water for 14 days at 4 and 25 °C, with 2-MIB concentration reduction by <10% and <16%, respectively. However, when tested in natural water, permanganate failed to prevent concentration reduction of 2-MIB because of the formation of manganese dioxide particles from the reduction of permanganate and 2-MIB adsorption on the particle surface. The concentration reduction was 15%–50% for the three tested water samples. Sodium hypochlorite successfully inhibited the reduction of 2-MIB from biodegradation at both temperatures. If the residual chlorine was higher than 0.5 mg/L during the 14 day experimental period, the concentration change in 2-MIB was less than 13% for all three tested natural water samples. The rate of 2-MIB degradation during the preservation may be described by first-order reaction, with the rate constants decreasing with increasing chlorine concentration. This study demonstrates that sodium hypochlorite may be used as an alternative chemical to prevent 2-MIB from biodegradation in water samples before analysis.

## Figures and Tables

**Figure A2:**
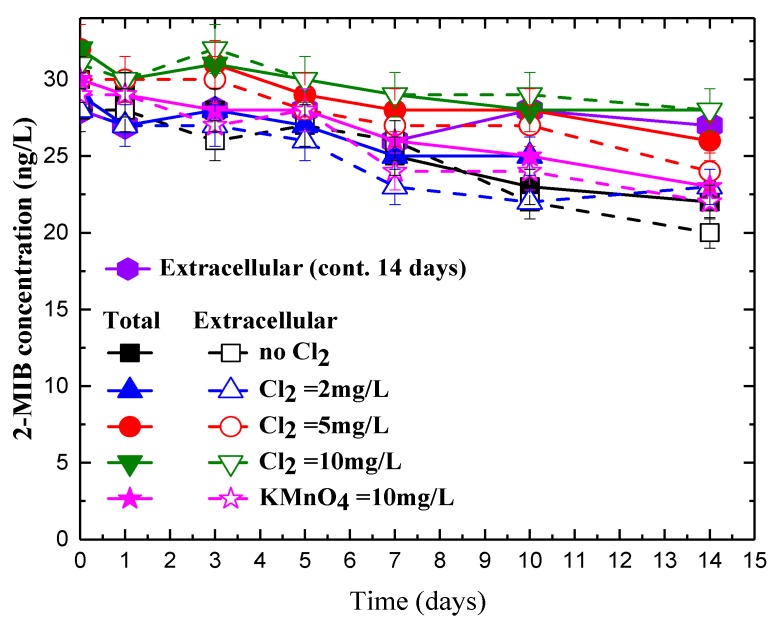
Changes of 2-MIB concentrations in the water samples for ZW reservoir preserved with chlorine and permanganate and stored at 4 °C.

## Appendix A

**Table A1:** The first-order degradation rate constants for 2-MIB in the three tested reservoir water. (Unit = day^−1^).

	Without Chlorine		Chlorine = 2 mg/L		Chlorine = 5 mg/L		Chlorine = 10 mg/L	
	Total	Extracellular	Total	Extracellular	Total	Extracellular	Total	Extracellular
AGD	7.36 × 10^−2^	5.29 × 10^−2^	3.78 × 10^−2^	3.52 × 10^−2^	1.21 × 10^−2^	9.61 × 10^−3^	1.10 × 10^−2^	1.20 × 10^−2^
	(R^2^ = 0.98)	(R^2^ = 0.83)	(R^2^ = 0.90)	(R^2^ = 0.75)	(R^2^ = 0.69)	(R^2^ = 0.56)	(R^2^ = 0.86)	(R^2^ = 0.86)
ZW	5.04 × 10^−2^	4.52 × 10^−2^	3.42 × 10^−2^	2.89 × 10^−2^	1.95 × 10^−2^	1.88 × 10^−2^	6.65 × 10^−3^	5.65 × 10^−3^
	(R^2^ = 0.99)	(R^2^ = 0.98)	(R^2^ = 0.94)	(R^2^ = 0.96)	(R^2^ = 0.94)	(R^2^ = 0.88)	(R^2^ = 0.60)	(R^2^ = 0.74)
NH	1.21 × 10^−1^	1.18 × 10^−1^	5.99 × 10^−2^	6.27 × 10^−2^	6.04 × 10^−3^	9.79 × 10^−4^	5.66 × 10^−3^	6.49 × 10^−3^
	(R^2^ = 0.97)	(R^2^ = 0.95)	(R^2^ = 0.98)	(R^2^ = 0.98)	(R^2^ = 0.72)	(R^2^ = 0.05)	(R^2^ = 0.57)	(R^2^ = 0.62)

## References

[1] Zhou X., Zhang K., Zhang T., Li C., Mao X. (2017). An ignored and potential source of taste and odor (T&O) issues-biofilms in drinking water distribution system (DWDS). Appl. Microbiol. Biotechnol..

[2] Jűttner F., Watson S.B. (2007). Biochemical and ecological control of geosmin and 2-methylisoborneol in source waters. Appl. Environ. Microbiol..

[3] Watson S.B., Ridal J., Zaitlin B., Lo A. (2003). Odours from pulp mill effluent treatment ponds: The origin of significant levels of geosmin and 2-methylisoborneol (MIB). Chemosphere.

[4] Suffet I.H.M., Corado A., Chou D., McGuire M.J., Butterworth S. (1996). Awwa taste and odor survey. Am. Water Works Assoc. (AWWA) Water Environ..

[5] Newcombe G., Morrison J., Hepplewhite C. (2002). Simultaneous adsorption of MIB and NOM onto activated carbon. I. Characterisation of the system and NOM adsorption. Carbon.

[6] Lin T.F., Liu C.L., Yang F.C., Hung H.W. (2003). Effect of residual chlorine on the analysis of geosmin, 2-MIB and MTBE in drinking water using the spme technique. Water Res..

[7] Bruchet A., Duguet J.P., Suffe I.H. (2004). Role of oxidants and disinfectants on the removal, masking and generation of tastes and odours. Rev. Environ. Sci. Biotechnol..

[8] Dionigi C.P., Lawlor T.E., McFarland J.E., Johnsen P.B. (1993). Evaluation of geosmin and 2-methylisoborneol on the histidine dependence of TA98 and TA100 salmonella typhimurium tester strains. Water Res..

[9] Srinivasan R., Sorial G.A. (2011). Treatment of taste and odor causing compounds 2-methyl isoborneol and geosmin in drinking water: A critical review. J. Environ. Sci..

[10] Wu J.-T., Jűttner F. (1988). Differential partitioning of geosmin and 2-methylisoborneol between cellular constituents in oscillatoria tenuis. Arch. Microbiol..

[11] Paerl H.W., Paul V.J. (2012). Climate change: Links to global expansion of harmful cyanobacteria. Water Res..

[12] Schöller C.E.G., Gűrtler H., Pedersen R., Molin S., Wilkins K. (2002). Volatile metabolites from actinomycetes. J. Agric. Food Chem..

[13] Börjesson T.S., Stöllman U.M., Schnűrer J.L. (1993). Off-odorous compounds produced by molds on oatmeal agar: Identification and relation to other growth characteristics. J. Agric. Food Chem..

[14] Dickschat J.S., Nawrath T., Thiel V., Kunze B., Müller R., Schulz S. (2007). Biosynthesis of the off-flavor 2-methylisoborneol by the myxobacterium nannocystis exedens. Angew. Chem. Int. Ed..

[15] Giglio S., Chou W.K.W., Ikeda H., Cane D.E., Monis P.T. (2011). Biosynthesis of 2-methylisoborneol in cyanobacteria. Environ. Sci. Technol..

[16] Izaguirre G., Taylor W.D. (2004). A guide to geosmin- and MIB-producing cyanobacteria in the united states. Water Sci. Technol..

[17] Kakimoto M., Ishikawa T., Miyagi A., Saito K., Miyazaki M., Asaeda T., Yamaguchi M., Uchimiya H., Kawai-Yamada M. (2014). Culture temperature affects gene expression and metabolic pathways in the 2-methylisoborneol-producing cyanobacterium pseudanabaena galeata. J. Plant Physiol..

[18] Sun D., Yu J., An W., Yang M., Chen G., Zhang S. (2013). Identification of causative compounds and microorganisms for musty odor occurrence in the huangpu river, china. J. Environ. Sci..

[19] Suurnäkki S., Gomez-Saez G.V., Rantala-Ylinen A., Jokela J., Fewer D.P., Sivonen K. (2015). Identification of geosmin and 2-methylisoborneol in cyanobacteria and molecular detection methods for the producers of these compounds. Water Res..

[20] Cook D., Newcombe G., Sztajnbok P. (2001). The application of powdered activated carbon for MIB and geosmin removal: Predicting pac doses in four raw waters. Water Res..

[21] Hsieh W.-H., Chang D.-W., Lin T.-F. (2014). Occurrence and removal of earthy-musty odorants in two waterworks in kinmen island, taiwan. J. Hazard. Toxic Radioact. Waste.

[22] Lalezary S., Pirbazari M., McGuire M.J. (1986). Oxidation of five earthy-musty taste and odor compounds. J. Am. Water Works Assoc..

[23] Nerenberg R., Rittmann B.E., Soucie W.J. (2000). Ozone/biofiltration for removing MIB and geosmin. J. Am. Water Works Assoc..

[24] Eaton R.W., Sandusky P. (2009). Biotransformations of 2-methylisoborneol by camphor-degrading bacteria. Appl. Environ. Microbiol..

[25] Izaguirre G., Wolfe R.L., Means E.G. (1988). Degradation of 2-methylisoborneol by aquatic bacteria. Appl. Environ. Microbiol..

[26] Lauderdale C.V., Aldrich H.C., Lindner A.S. (2004). Isolation and characterization of a bacterium capable of removing taste- and odor-causing 2-methylisoborneol from water. Water Res..

[27] Ho L., Hoefel D., Bock F., Saint C.P., Newcombe G. (2007). Biodegradation rates of 2-methylisoborneol (MIB) and geosmin through sand filters and in bioreactors. Chemosphere.

[28] Oikawa E., Shimizu A., Ishibashi Y. (1995). 2-methylisoborneol degradation by the cam operon from pseudomonas putida ppg1. Water Sci. Technol..

[29] Kim T.-K., Moon B.-R., Kim T., Kim M.-K., Zoh K.-D. (2016). Degradation mechanisms of geosmin and 2-MIB during UV photolysis and UV/chlorine reactions. Chemosphere.

[30] Tung S.C., Lin T.F., Liu C.L., Lai S.D. (2004). The effect of oxidants on 2-MIB concentration with the presence of cyanobacteria. Water Sci. Technol..

[31] Hung H.-W., Lin T.-F., Chiu C.-H., Chang Y.-C., Hsieh T.-Y. (2010). Trace analysis of *n*-nitrosamines in water using solid-phase microextraction coupled with gas chromatograph–tandem mass spectrometry. Water Air Soil Pollut..

[32] Chen Y.-M., Hobson P., Burch M.D., Lin T.-F. (2010). In situ measurement of odor compound production by benthic cyanobacteria. J. Environ. Monit..

[33] Tian X.Y., Han X.K., Huang Z.H. Experimental study on combing chlorine and potassium permanganate to inactivate bacteria. Proceedings of the 2010 4th International Conference on Bioinformatics and Biomedical Engineering.

[34] Tucker C.S., Boyd C.E. (1977). Relationships between potassium permanganate treatment and water quality. Trans. Am. Fish. Soc..

[35] Freitas R.M., Perilli T.A.G., Ladeira A.C.Q. (2013). Oxidative precipitation of manganese from acid mine drainage by potassium permanganate. J. Chem..

[36] Moyers B., Wu J.S. (1985). Removal of organic precursors by permanganate oxidation and alum coagulation. Water Res..

[37] Zawacki J. (1992). KMnO_4_ contributes to least cost treatment solution. Water Eng. Manag..

[38] Rand J.L., Gagnon G.A., Knowles A. (2014). Establishing minimum free chlorine residual concentration for microbial control in a municipal drinking water distribution system. Water Pract. Technol..

[39] Kaleli H.A., Islam M.R. (1997). Effect of temperature on the growth of wastewater bacteria. Toxicol. Environ. Chem..

